# Individualised Responsible Artificial Intelligence for Home-Based Rehabilitation

**DOI:** 10.3390/s21010002

**Published:** 2020-12-22

**Authors:** Ioannis Vourganas, Vladimir Stankovic, Lina Stankovic

**Affiliations:** Department of Electronic and Electrical Engineering, University of Strathclyde, Glasgow G1 1XW, UK; vladimir.stankovic@strath.ac.uk (V.S.); lina.stankovic@strath.ac.uk (L.S.)

**Keywords:** accountable artificial intelligence, responsible artificial intelligence, transparent artificial intelligence, hybrid ensemble learning, home-based rehabilitation, patient-centric individualised rehabilitation, automated timed up and go test, automated five time sit to stand test

## Abstract

Socioeconomic reasons post-COVID-19 demand unsupervised home-based rehabilitation and, specifically, artificial ambient intelligence with individualisation to support engagement and motivation. Artificial intelligence must also comply with accountability, responsibility, and transparency (ART) requirements for wider acceptability. This paper presents such a patient-centric individualised home-based rehabilitation support system. To this end, the Timed Up and Go (TUG) and Five Time Sit To Stand (FTSTS) tests evaluate daily living activity performance in the presence or development of comorbidities. We present a method for generating synthetic datasets complementing experimental observations and mitigating bias. We present an incremental hybrid machine learning algorithm combining ensemble learning and hybrid stacking using extreme gradient boosted decision trees and *k*-nearest neighbours to meet individualisation, interpretability, and ART design requirements while maintaining low computation footprint. The model reaches up to 100% accuracy for both FTSTS and TUG in predicting associated patient medical condition, and 100% or 83.13%, respectively, in predicting area of difficulty in the segments of the test. Our results show an improvement of 5% and 15% for FTSTS and TUG tests, respectively, over previous approaches that use intrusive means of monitoring such as cameras.

## 1. Introduction

Modern healthcare and socioeconomic reasons in the post-COVID-19 world require home-based rehabilitation without specialist assistance. As discussed in detail in [1], the need for home-based rehabilitation is multifaceted, from patient psychology to reduced hospital funding per patient. Approximately 50,000 patients are discharged for home-based rehabilitation every year in England alone [2].

According the review in  [1], patient motivation and engagement have a critical impact on the success [3] of home-based rehabilitation, as there are no frequent checks from a supervising person. Many current approaches are abandoned within the first 90 days of use because the lack motivating and engaging user interfaces [4]. The motivation is linked to goal setting and it depends on individual achievements [5]. Thus, the technology must be co-created with the end user to both adapt to the individual and provide relevant and achievable goals through a dialogic approach to simple motivational user feedback [1].

A detailed technological review of home-based rehabilitation technologies [6] concludes that current existing technologies provide generic, “one size fits all” solutions—instead of being tailored to specific user needs, leading often to poor motivation and the user’s quick loss of interest, but also sometimes unfair and biased outcomes. Thus, there is an increased need for new approaches that combine artificial ambient intelligence (AmI) and individualisation to support engagement and motivation in home-based rehabilitation [6] that also comply with ethical principles and that incorporate accountability, responsibility, and transparency. The latter is particularly important to improve trust through being able to explain to technology providers and end users the decisions made by the AmI. This explanation is necessary for the acceptance of motivational feedback and to provide user awareness and an understanding of the individual goals set by the technology against their progress.

Existing AmI solutions often introduce assumptions and bias of the developer in the decisions taken by the algorithm [7]. This issue is reflected in recent efforts for ethical Artificial Intelligence (AI) requirements and design practices, which highlight a deeper underlying consideration regarding the used values and norms, and their reinforcement through the evolution of the machine’s autonomy [8]. Thus, there is an urgent global need for accountable [9], responsible [10], and transparent [11] artificial intelligence (ART AI) to enable wider use of AI [12] based on generally acceptable value systems [13].

Responsible AI models consider user practices, value systems, ethics, and implications for various communities. They are based on an approach which is ethically sound, regulation compliant, governed, robust, unbiased, fair (just), interpretable, explainable, and secure (non-maleficent) [14].

In this paper, we discuss a design of a patient-centric individualised, home-based rehabilitation support system focusing on the following aspects of ART AI:

1. Unbiased AI: A significant body of work has contributed to methods for bias-free machine learning (ML) models [15,16,17]. We aim to follow state-of-the-art bias reduction approaches [18] in our proposed rehabilitation support approach based on designing an appropriate and balanced training dataset that would remove bias due to class under-representation.
2. Explainable AI: There has been strong research interest in the field of explainable AI (XAI) in recent years [19]. However, these approaches are mostly focused on the social aspect (user response to XAI) [11], or the implications of manipulating inputs to generate false negative or false positive responses [20].
3. Interpretable AI: Interpretable and justifiable outcomes of AI require transparency of the ML model as well as the model’s decisions and behaviours [21]. Current EU regulation allows for individuals to enquire about AI decisions [22]. However, regulation is not well defined for the design and development of such models, particularly in the medical applications domain [23]. Moreover, accountability and transparency are strongly interlinked with interpretability [17].

Unbiased AI is particularly significant for home-based rehabilitation systems, as they are used by a variety of users including elderly and young, male and female, and of varied height and weight, and all need to receive the same and fair level of support. Explainability and interpretability are also key to enable user engagement as we have identified in [6] and strongly link to complexity and motivation. In this work, we will focus on the information the model can generate to provide further insight to the decision-making process, establish a dataset to address the bias issue, and aim for interpetability to address some of the challenges in home-based rehabilitation AI.

In summary, the contributions of this paper are:

- Methodological steps to produce a new synthetic dataset based on statistical clinical results reported in the literature for training ML algorithms to avoid bias and ensure fairness in autonomous system outcomes (Section 3.1.2);
- A novel hybrid ML algorithm to meet the individualisation, interpretability, and ART design considerations while maintaining a low computational footprint (Section 4);
- Interpretability of the designed solution, including feature importance for a patient-centric individualised, responsible home-based rehabilitation support (Section 4.1);
- A detailed simulation performance comparison and analysis demonstrating that the proposed approach outperforms existing work, used as benchmark, by 5% for FTSTS and 15% percent for TUG test (Section 5 and Section 6).

A detailed review of the methodology, performance, and limitations of current home-based rehabilitation digital technologies is provided in [6]. Next, we focus on ART artificial ambient intelligence approaches in Section 2 and identify the state-of-the-art as well as further improvements required. We investigate and present a system to address them in Section 3 and Section 4. The evaluation of the proposed system along with the results is presented in Section 5, followed by the discussion in Section 6. Finally, conclusions and the identified future work are presented in Section 7.

## 2. Literature Review of ART AI and Its Applicability to Home-Based Rehabilitation Systems

In medical applications such as rehabilitation, the requirement for ART AI is heightened. High-quality data as well as the responsibilities of curation, provenance and bias avoidance are critical [23]. As mentioned previously explainability and interpretability are important for engagement but also help avoid unintended consequences. In the medical domain, unintended consequences of AI can be significantly damaging or irreversible [23]. The benefit to patient care and society, however, can be tremendous. Thus, ART solutions are of paramount importance.

In [24], the need for explainability and interpretability in medical applications is discussed. It is found that deep learning in particular is often difficult to interpret and explain. Additionally, incorporating medical knowledge in the design of ML solutions is important to generate XAI. Outputs of XAI should be in human legible terms such as sentences of text, and embedded optimisations and decisions should be explainable. Additionally, ref. [25] suggest that feedback in different forms must be provided and that the ART AI adapts not only the model to the user but also the form of the provided output. Thus, individualisation and layered/selectable feedback is necessary for the non-technical user of AI. However, these systems refer to the medical professional and are not targeting the home-based rehabilitation environment or the end user. According to [26], the target user audience is a key consideration for the XAI design. Additionally, the authors discuss the explainability of well-known models. We will refer to their taxonomy as we analyse the suitability of various models throughout this paper with a focus on the end user of the home-based rehabilitation system.

Furthermore, according to [27], AmI are the most appropriate autonomous candidate systems for responsible AI-driven tele-rehabilitation because they have the opportunity to provide dialogic explanations of the system’s behaviour and to observe the user’s explanation needs. Indeed, AmI systems are by nature embedded into the environment, they are context-aware, they can be individualised, and most importantly, they can change in response to the user and can anticipate the user’s desires. This relates to goal setting, motivation enhancement, and engagement, which are primary factors affecting home-based rehabilitation [6].

Home-based rehabilitation methods must also be capable of addressing the challenges of reduced computational cost and physical constrains of the IoT devices. Recent studies have improved computational efficiency of advanced AI methods and proved applicability in AmI scenarios. For example, random decision forests in general have proven to be an effective AI approach for medical applications [28] at reasonable complexity. Recently, ensemble methods but also hybrid methods that combine heuristic algorithms with heterogeneous ML models have improved performance with various effects on computational footprint [29]. To further reduce computational footprint, in [30], XGboost (originally presented in [31]) is used as part of the proposed ensemble learning model. However, the solution is restricted to single-feature data stream learning. The approaches mentioned above are promising in terms of accuracy, complexity, and computational footprint, and will be investigated further in the next section for suitability in our specific problem domain in Section 3.

Deep learning approaches are, in general, considered complex, as they require large computational resources and large datasets, and hence are often unsuitable for light home-based rehabilitation applications. However, Keras API, one of the most popular platforms to easily implement deep learning models, offers efficient deployment of neural networks (NNs) for complex multi-dimensional problems [32,33], and could be suitable for prediction on IoT devices. However, according to [26], NNs are not interpretable or explainable because of the black-box problem and the complexity of the algorithms. Moreover, they require significant computational capacity to train the models.

The approach of [34] utilises ensemble learning as well as edge computing for security in IoT applications in real time, demonstrating the low computational footprint of gradient boosting algorithms. Moreover, for some applications which require an increased level of privacy and security, federated learning could also be suggested [35]. In terms of ART AI, boosted trees can provide outputs to support explainability of ML models in an IoT context [26,36]. Furthermore, boosted trees improve the bias variance trade-off [37]. However, ref. [26] suggests that ensembles of trees require further simplification to relay information, and make them the most appropriate approach for further investigation to improve the ART component of ensemble learning approaches.

Further improvements can be achieved by combining boosting (XGBoost) and stacking with *k*-nearest neighbour (*k*-NN) on a server [38] while at the same time improving the robustness and reducing overfitting issues. According to [26], *k*-NN is also easy to interpret with no further post hoc analysis requirements. However, the stacking approach in [38] has a significant training overhead that would make individualisation impossible in an AmI scenario due to computational capacity restrictions.

In terms of patient evaluation for home-based rehabilitation progress monitoring, two medical tests are identified in [1]. These are the Timed Up and Go (TUG) and the Five Time Sit To Stand (FTSTS) tests. The state-of-the-art AI methods for TUG and FTSTS focus on the use of cameras or wearable sensors. For example, in [39], wearable sensors are used with naive Bayes as well as NN classifiers for TUG, where NNs, being the most performant, only reported 85% accuracy. In [40], the Kinect sensor is used for FTSTS with *k*-NN and decision trees with about 90% accuracy. In [41], wearable IMU sensors, Kinect, and a camera are used with a boosted decision tree, resulting in accuracy of 94.68%. A review of similar publications presented in [41] demonstrates that this is the state of the art for FTSTS AI models in terms of accuracy. Thus, according to the engagement and motivation design criteria in [6], the state-of-the-art approaches using AI fail in terms of acceptance because non-wearable and non-intrusive methods are preferable. Moreover, none of these approaches address individualisation or the ART requirements and do not provide feedback designed for motivation and engagement.

In summary, the state-of-the-art approaches in TUG and FTSTS AI are not designed based on the user’s requirements. Existing AI approaches that ensure high accuracy, such as deep learning, do not meet the ART AI requirements and do not maintain low computation footprint suitable for individualised incremental learning on AmI systems to support home-based rehabilitation. On the other hand, XGBoost can lead to high accuracy and low computation footprint while providing some level of transparency. Hybrid methods can further improve accuracy and have a variable impact on computation footprint depending on implementation.

Motivated by the low computational cost of boosting, in this paper, we investigate ART for AmI in the home rehabilitation systems and propose a low computational footprint boosting and hybrid stacking ensemble learning method to deliver intelligent and individualised ART AI to meet the criteria presented in [6], where, following a rigorous review of technologies for home-based rehabilitation, we identified the requirements for engagement and motivation enhancing technologies for home-based rehabilitation. In particular, we combine XGBoost and *k*-NN algorithms without the added overhead of stacking on a computational power restricted setup. Both models are categorised as transparent with few additional post hoc analysis steps necessary to address explainability [26].

### ART Design Considerations

To design our system in accordance with the ART AI principles, we have reviewed design time considerations. Responsible AI ethics can be categorised in three groups [42]: ethics by design (technical/algorithmic), ethics in design (regulatory/engineering), and ethics for design (code of conduct/standards). In every case, the design time considerations for the development of ethical AI models is highlighted. These categories include the ethical reasoning capabilities integrated in the model’s behaviour, the analysis and evaluation of ethical implications in social structures, and ensuring integrity of the developers and users.

Design time considerations have recently been published for European Research and Innovation that apply to safety-critical systems with AI [43]:

1. avoid bias and prejudice in training data, or make biases clear to user population;
2. ethical principles embedded into AI development;
3. interdisciplinary teams are crucial;
4. transparent data provenance (input, output);
5. lay people need to understand AI decisions;
6. decision justification.

We follow these design time considerations in the development of the proposed methodology and the design of the proposed hybrid ML approach for individualised ART-driven rehabilitation. We discuss our ART AI design approach and address the six design time considerations for our proposed system in Section 4.1.

## 3. Methodology

In this section, first we describe the experimental dataset used, and then provide a methodology for generating a synthetic dataset to ensure unbiased decision-making. Then, we evaluate well-known machine learning methods on both datasets. Finally, we propose a hybrid learning approach to mitigate limitations of the existing solutions.

### 3.1. Training Dataset Design

To design responsible-AI driven home-based rehabilitation system, we first need to generate, collect and prepare an appropriate dataset. We require a dataset that can be used both to monitor progress of activities relevant to rehabilitation and also to diagnose both individual difficulties and medical conditions or comorbidities. We leverage on the small experimental dataset of [1], where a sensor-based platform was introduced for data collection and analysis of the two medical tests used in subject evaluation, namely TUG and FTSTS. A thorough review of patient evaluation medical tests and their relation to home-based rehabilitation is presented in [1].

The two tests were selected as they are relevant to a variety of activities of daily living, can be performed without medical supervision and in the home environment, and have been used to evaluate the progress and condition of post-stroke patients in line with the criteria presented in [6]. Both tests evaluate lower limb strength, mobility, static and dynamic balance, functionality, and durability, all of which are relevant to rehabilitation outcomes. Improvement in performing the tests over time translates to better ability to perform daily tasks (such as sitting and standing, walking small distances), self-efficacy, and engagement with rehabilitation as discussed in [1]. Additionally, the use of medical tests provides the benefit of evaluating the system against medical standards and clinical specification. However, to achieve high accuracy, large datasets representative of real patient measurements are needed, which, to the best of our knowledge, are not publicly available for the problem mentioned above.

A poorly designed dataset used for training the models can easily lead to biased or inaccurate outcomes [12]. To mitigate this, intrinsic, contextual, and representational data quality dimensions as presented in [44] should be considered when designing a training dataset. Specifically, to generate a synthetic dataset and address the appropriate amount and quality of data, we consider carefully the accuracy, believability, objectivity, and reputation through developing a method that generates data from published medical research outputs.

To generate a new synthetic dataset that will lead to high accuracy and objectivity, we start from the experimental data from [1], and augment it using statistical published data, collectively presented in [45] for TUG and [46] for FTSTS tests. We elaborate on the datasets next.

#### 3.1.1. Experimental Dataset

In [1], data on completion of TUG and FTSTS tests were collected through the presented low-cost home rehabilitation system for eight participants. The participants simulated difficulties in the various stages of the TUG test simulating elderly individuals performing the same test. We consider each difficulty level as a label for the purposes of this dataset. The features recorded for each participant are:

1. Test completion time (seconds)
2. Age (years)
3. Height (meters)
4. Weight (kg)
5. Body mass index (BMI) (kg/m${}^{2}$ calculated using Equation (1))
6. Sex (male/female)

(1) $$\mathrm{BMI}=\frac{\mathrm{Weight}\;(\mathrm{kg})}{{[\mathrm{Height}\;\left(m\right)]}^{2}}$$

The use of BMI, in addition to height and weight, could improve the prediction accuracy. Indeed, according to [47], the inclusion of features which are generated from other features (using linear or polynomial equations) can improve the performance of more complex models such as NNs. We will investigate the influence of features such as BMI in Section 5.

This dataset had no missing, malicious, erroneous, inconsistent, or irrelevant data points. Data formatting was necessary to map gender and category data entries from String to Integer values. Some outliers were present in the originally recorded data as evident in [1]. These outliers were removed following the >3σ (σ denotes standard deviation) approach [48]. Finally, all measurements were normalised in the range [−1,1].

The categories (classes) that we have recorded for TUG were: (a) difficulty to walk, (b) difficulty to turn, (c) difficulty to stand/sit, (d) normal, and (e) fast. For FTSTS, the classes are: (a) difficulty to stand/sit annotated as slow, and (b) fast.

There are two major issues with this experimental dataset. First, it is small—only eight participants are included, which will lead to inaccurate outcomes. Second, the dataset is unbalanced, potentially leading to bias and affecting the objectivity requirement. Indeed, first, the class normal in TUG (and fast in FTSTS) has fewer collected data points.

Class unbalance is addressed with the use of the synthetic minority over sampling technique (SMOTE) [49]. With the use of SMOTE, all classes were balanced, with each class having 40 datapoints both in TUG and FTSTS. In total, 17 samples were generated by SMOTE for TUG, of which one was for the fast and 16 for the normal class. Moreover, 7 were generated for FTSTS for the difficulty/slow class.

Besides class unbalance, the dataset suffers from data imbalance; the resulting dataset had 87% male participant entries and all participants belonged to the 20–45 years old age group, clearly leading to biased outcomes. To identify the effect of this bias, we will discuss the feature importance for the proposed method in Section 4.

#### 3.1.2. Synthetic Dataset

ML models have demonstrated success in many application fields. However, since advanced ML models are data-driven; to translate these successes to medical fields, it is necessary to provide large datasets to develop and train the models. Generating an open-access datset based on collected data is a significant obstacle due to patient data privacy and cost [50]. Hence, the rehabilitation field suffers from a lack of appropriate datasets to develop and test advanced ML models. In many other fields, where real data collection is expensive or impractical, synthetic datasets are generated and employed. However, the medical domain has been reluctant to embrace synthetic datasets.

Most recently, the community has recognised this adverse effect [50,51]. In [51,52] the main arguments against synthetic datasets are rebutted through a comparative analysis of actual and synthetic datasets used to train and test the model. It is demonstrated that the synthetic dataset can produce equal performance if synthesised from a statistical representation of the actual cohort of patients. In [50], it is discussed that if the model performs accurately when tested with unseen real patient data, then the synthetic dataset can be accepted as representative of the reality. In this paper, we follow this line of work; we synthesise a dataset, ensuring statistical agreement with real measurements, and test the trained model using experimental dataset in Section 5.

The reasons for synthesising a dataset are:

1. To meet the requirements of [6] for transferability and co-morbidity diagnosis, the underlying reason being the development of co-morbidity for stroke survivors and early diagnosis/warning to carers;
2. To improve variance and bias of our experimental dataset;
3. To improve accuracy of our model;
4. To include a wider range of height, weight, age and BMI that is representative of the wider population that is dischared to home-based rehabilitaiton directly impacting bias;
5. To improve believability by sourcing information from medical journals, where a larger cohorts of geriatric subjects and patients have participated in experiments;
6. To improve objectivity by including information from experiments with participants diagnosed to have the medical conditions that can be developed as co-morbidities, such as Parkinson’s and dementia.

It is important to highlight here that the synthetic dataset will be used for initial training of the model. However, as one of the criteria in [6] is to provide individualised solutions, our model would be constantly re-trained and corrected using real data acquired by the user of the device presented in [1]. Thus, throughout the system lifetime, the synthetic data will eventually be a proportionally small contributor to the model.

Furthermore, the synthetic dataset could not be generated from the experimental dataset using an oversampling method. SMOTE could not be used in this case as (1) experimental dataset is gender- and age-unbalanced; (2) SMOTE requires an input dataset and the original dataset, described in the previous subsection, does not link the six features with specific conditions. Thus, an alternative approach for dataset synthesis is proposed next.

We consult a database of medical/clinical research publications that present statistical results of experiments with patient cohorts for TUG and FTSTS. Each publication states the medical condition of the cohort, the number of subjects and the statistical characteristics of the cohort. To the best of our knowledge, this database presents a comprehensive review of all medical conditions for which TUG and FTSTS are used to evaluate patients. Every condition reviewed in [45,46] was included for TUG and FTSTS, respectively. Publications cited in [45,46] were included as inputs in the synthetic dataset algorithm, if they presented statistical descriptors for all of the features used in the experimental dataset above, or if the features could be extrapolated or calculated (e.g., BMI). Publications were in turn excluded if any of the features was not reported or could not be extrapolated from the information presented. Because the selection is not made using a specific feature as the criterion, this selection method does not introduce a particular bias to the pool of included publications. Additionally, publications were not excluded based on race, sex, or ethnicity of the participants.

In addition to the difficulty classes mentioned in the experiment dataset for TUG, the following condition classes were finally considered:

Healthy, Geriatric, Parkinson’s, Parkinson’s non-fallers—medication, Parkinson’s non-fallers—no medication, Parkinson’s fallers, Dementia mild/moderate, Dementia severe, Arthritis improvement, Arthritis knee arthroplasty, Arthritis, Stroke, Brain injury, Bilateral vestibular hypofunction, Unilateral vestibular hypofunction, Spinal injury, Paraplegia, Tetraplegia. In total, 16 publications remained for TUG based on the inclusion criteria, namely [53,54,55,56,57,58,59,60,61,62,63,64,65,66,67,68,69]. A total cohort of n=937 subjects is represented by these publications with age in the range [5,112] years, height in the range [0.81,2.20) m, and weight in range (30,136) kg. Each condition class has 280 datapoints and the overall set has a total of 5040 datapoints.

Similarly, for FTSTS, the condition classes are: Healthy, Geriatric, Geriatric fallers, Parkinson’s stage 1, Parkinson’s stage 2, Parkinson’s stage 2.5, Parkinson’s stage 3, Parkinson’s stage 4, Parkinson’s, Arthritis, Arthritis knee arthroplasty, Stroke, Vestibular disorder. In total, 12 publications remained for FTSTS based on the inclusion criteria, namely [56,70,71,72,73,74,75,76,77,78,79,80], while 3 were excluded. A total cohort of n=2381 subjects is represented by these publications with age range (11,93] years, height in the range [0.94,2.35) m, and weight in range (22,120) kg. Each class has 240 datapoints and the overall set has a total of 3120 datapoints.

**Figure 1:**
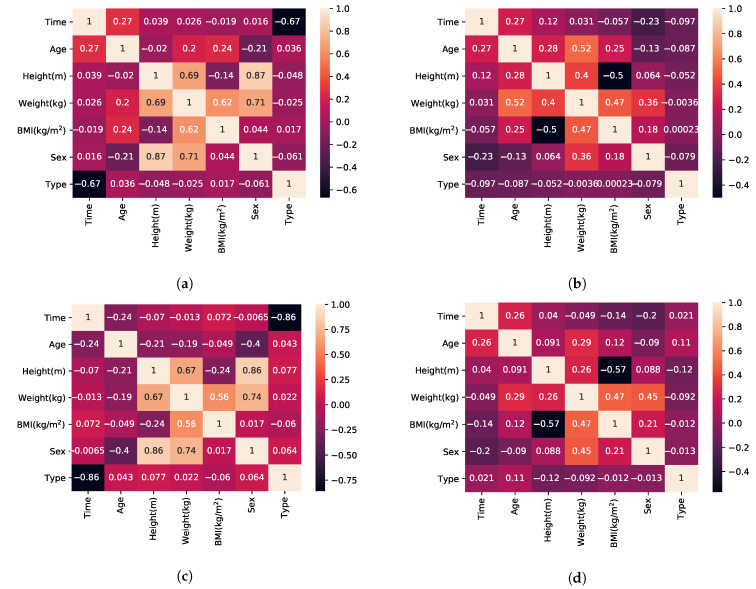
(**a**) Correlation matrices for Timed Up and Go (TUG) experiment, (**b**) TUG synthetic data, (**c**) Five Time Sit To Stand (FTSTS) experiment, and (**d**) FTSTS synthetic data.

According to [73], BMI has low correlation to the FTSTS test completion time (*p*-value =0.4) so higher variability was introduced compared to the TUG synthetic dataset as reflected in Figure 1. The same is true for height [75,80]. On the contrary, hand positioning has a significant correlation to completion time. All participants in the experiments and included publications followed the same hand positioning, hands crossed over chest. This parameter was ignored [81], given that it would be the same value for all datapoints and hence provided no difference in completion time.

Algorithm 1 is proposed to generate synthetic data points from the statistical properties presented in considered publications, where μ and σ denotes class mean and standard deviation. The algorithm is based on the following observations:

- There is a linear relationship between time of completion and age in both TUG and FTSTS tests [68,71], but there are several exceptions to this rule, so variance is required to represent a more realistic relationship.
- In [82], an almost linear relationship is presented between age and BMI for adults over 40 years of age. This is further supported by the widely accepted BMI charts [83]. However, it is not absolute, so variability is again required.
- BMI, by definition, has a linear relationship to weight (Equation (1)).
- According to [58] female subjects perform faster than male subjects in TUG, which is a superset of activities in relation to FTSTS.
- Sex and weight also have a relationship that is linear in regards to the mean weight value of the population with higher variance (wide standard deviation causing overlap between the two populations) [84].
- Height is calculated using Equation (1) after the pair {BMI, weight} has been established.
- Sex and age have no correlation (observation supported by our experimental dataset and the mean age value reported for each sex in each of the included publications).

These observations are further supported by the Experimental dataset observations both in terms of linearity and variability. Algorithm 1 presents the relevant implementation.

To introduce linearity, e.g., to ensure a linear relationship between time of completion and age, we generate pseudo-random numbers from the Gaussian distribution of each feature and generate a sorted sequence (function sort in Algorithm 1). Because lists of all features are sorted from lower to higher, the linear relationship is generated; for example, the lowest completion time will be aligned to the lowest age. Then, to introduce variability, we swap the order of several data points in one of the two feature columns (function insertVariability in Algorithm 1)—for example, keeping completion time sorted and swapping values in the age column. If variability is required between one feature and a set of other features, then the same swaps are applied to the full set. For example, if we swap data point 3 with data point 7 in BMI to introduce further variability compared to age, we apply the same swap to height and sex as well, thus maintaining the correlation between the BMI, weight, and gender sequences.
**Algorithm 1** Generate features for *n* datapoints of Class *x*.**Require:** $n,x,\mu_{time},\sigma_{time},\mu_{age},\sigma_{age},\mu_{bmi},\sigma_{bmi}$**Require:** $\mu_{weight},\sigma_{weight},\mu_{height},\sigma_{height},{\%}_{female}$ $time\leftarrow sort(X∼N\left(\mu_{time},\;\sigma_{time}^{2}\right))$ $age\leftarrow sort(Y∼N\left(\mu_{age},\;\sigma_{age}^{2}\right))$ age←insertVariability(Y) $bmi\leftarrow sort(Z∼N\left(\mu_{bmi},\;\sigma_{bmi}^{2}\right))$ bmi←insertVariability(Z) $weight\leftarrow sort(W∼N\left(\mu_{weight},\;\sigma_{weight}^{2}\right))$ $gender\leftarrow S_{i}=0\;\forall i\in \left\{0,n*{\%}_{female}\right\}\wedge S_{i}=1\;\forall i\in \left\{n*{\%}_{female}+1,n\right\}$ {bmi,weight,gender}←insertVariability({Z,W,S}) $height\leftarrow H=\sqrt{\frac{W}{Z}}$**Ensure:** $\mu_{height}=\mu_{H}\;\mathrm{and}\;\sigma_{height}=\sigma_{H}$

Finally, to verify the validity of the generated datasets for FTSTS and TUG, we compare the correlation matrix of the features of the synthetic datasets to the original recorded experimental datasets. We used the correlation matrix as a guide for the amount of variability to be introduced. The final correlation matrices are presented in Figure 1.

It is evident that a similar correlation matrix emerges in both the experimental and the synthetic datasets. There is one significant difference. Sex is highly correlated with both height and weight in the experiment. This is because the only female participant in the experiments was also the shortest and lighter. This generated a bias between height and sex, and BMI results also represent an unrealistic correlation to height. Thus, the sex bias is the most important bias in the case of the experiment. Addressing this by introducing more female subjects with different height, weight, and BMI measurements would improve all of the biases resulting from the bias towards male participants. The synthetic dataset, on the other hand, demonstrates a weaker relationship between sex and height as well as sex and weight and height with all other features, including BMI, which is closer to reality and thus compensating for the biases in the experimental dataset.

**Figure 2:**
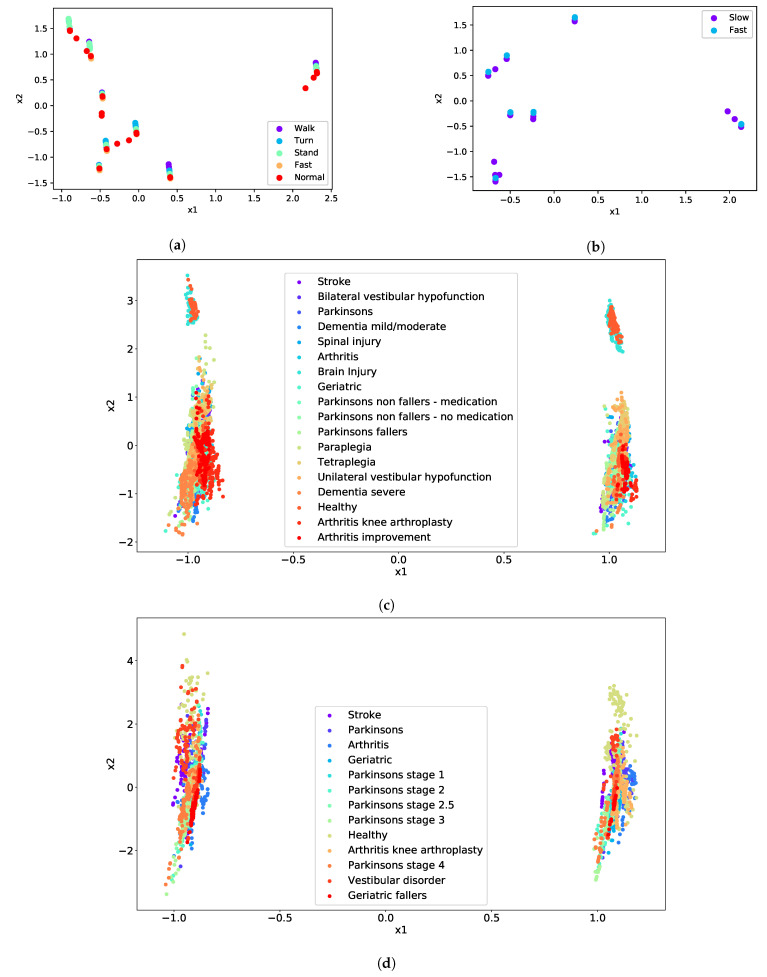
(**a**) Principal component analysis biplots for TUG experiment data, reducing the features to 2, (**b**) FTSTS experiment, (**c**) TUG synthetic, and (**d**) FTSTS synthetic.

Figure 2 presents both the datasets after projecting all the features to two dimensions using PCA. Visualising the data also demonstrated a potential polynomial function as displayed in Figure 2a,c where all the points seam to follow a polynomial curve with different offset for each of the classes. This relationship is further investigated in the following section.

### 3.2. Evaluation of ML Methods for Supervised Classification

Here, we review the ML models that are most relevant to the home-based rehabilitation problem. Then, we implement those and compare their accuracy to identify the most promising models for further investigation and development.

We focus on the problem of classifying the patients into the classes defined in Section 3.1, independently considering TUG and FTSTS datasets. As our data were labeled both for the experiment and the synthetic datasets, we have focused on supervised learning methods.

The design requirements of AmI systems imposed restrictions: first, through the computational capabilities/capacity of the AmI and second, through the available programming language with sufficient ML libraries and the low-level programming interface to collaborate with the sensors and components of the system. As a result Python was the selected programming language, with the pandas library for numerical analysis and dataset representation, sklearn library for ML and classification support, and low-level libraries for communication protocols used by the AmI system.

NNs were not evaluated further as they did not meet the ART AI requirements and also the individualisation requirements on a resource-constrained AmI system. As discused in Section 2 computational capacity requirements for training, and thus re-training, would be prohibitive for deployment on AmI [85]. Furthermore, convolutional NNs have performed similarly to XGboost in terms of accuracy in non-imaging applications [86,87]. XGboost has produced models of high accuracy in a variety of problems when compared to NNs [88]. Thus, accuracy is not being sacrificed in favour of explainability and computation constraints.

In [89], a review of all classification algorithms available in Python libraries is presented along with their computational requirements and effect on bias and variance. Based on this review, the most relevant classifiers to our problem specification and multi-class classification in the sklearn library are support vector machine (SVM) and XGBoost for the following reasons. SVMs are powerful yet lightweight in terms of computational requirements. We use the default one-versus-all multi-class classification approach. On the other hand, XGBoost can mitigate variance and bias in datasets while automatically correcting model biases through the random forest underlying approach. This also results in reduced risk of over-fitting. XGBoost operates on a one-versus-all principle but optimises the models based on softmax probabilities.

With a relatively small number of features (seven), we first implemented simpler approaches such as SVM and polynomial regression. All possible kernels were tested with the SVM including linear, polynomial of degree 4, rbf, and sigmoid. In all cases, the regularisation parameter C=1, which is the default value in sklearn Python library. The best performing kernel was the linear and only the accuracy of this kernel is discussed in this section. However, the subtle correlations between the features as well as the sheer number of classes in the synthetic dataset proved challenging for these simpler approaches, as demonstrated by the low accuracy in Table 1. For the regression approach, the Python OLS multiple regression algorithm was used [90]. Both linear SVM and the regression approach are excluded from further hyper-parameter tuning because of their accuracy being <40% in all multi-class cases.

Higher complexity models were then tested. They were selected because they have low computation requirements for the prediction phase and were appropriate for the ambient intelligence hardware developed in our experiments [1]. XGboost was initially set up with maximum depth equal to double the number of classes and the multi:softmax objective function, which are required settings for multi-class problems. Additionally, for an initial evaluation of the *k*-NN model, k=5 nearest neighbours was used as an even number that respects the low computation requirements and is a starting point to be further optimised. As these methods reported higher accuracy, further hyper-parameter tuning is undertaken in the next section. The accuracy of all the models was evaluated using an 80% training and 20% testing dataset split and fivefold cross validation [91]. Note that it is shown in [92] that a number between 5 and 10 folds usually provides similar results.

Table 1 presents the accuracy score of the model (all classes) averaged across the fivefold cross validation. This table demonstrates the accuracy of each model for the particular dataset with the purpose of identifying which model would be most suitable for further tuning and evaluation. XGBoost and *k*-NN have a significantly better accuracy in all datasets compared to all other models. Additionally, XGBoost decision trees and *k*-NN are easier for humans to understand when many features are involved [36] with little or no post hoc analysis [26] as discussed in earlier sections. Moreover, the XGBoost Python implementation can provide results such as the probability of each cluster, and the decision weights used for each tree to improve transparency and support interpretability and explainability and will be further discussed in Section 4.

Differences in the results between the experiment and synthetic datasets presented in Table 1 can be explained by either the increased bias of the experiment dataset in the TUG case or the larger number of classes (5 and 2 in experiment dataset, versus 18 and 13 in TUG and FTSTS datasets, respectively) and higher number of data points in the synthesised dataset in the FTSTS case. Indeed, as a result of bias (see Figure 1), the classification error on the experiment dataset is generally higher for TUG (e.g., TUG experiment *k*-NN versus TUG synthetic *k*-NN results). For FTSTS, however, the increased number of classes (e.g., 13 for FTSTS synthetic versus 2 for FTSTS experiment) can lead to higher probability of misclassification affecting accuracy of classifiers when applied to synthetic datasets (e.g., FTSTS experiment XGBoost versus FTSTS synthetic XGboost results). Finally, for TUG and FTSTS, the difference between experiment and synthetic dataset must be discussed in terms of dataset size. Experiment datasets are smaller datasets, and this has a profound effect on accuracy. In summary, experiment dataset results are negatively affected by bias and a small number of training samples. However, the results on the synthetic dataset demonstrate that similar or better accuracy can be achieved with well-designed dataset, even with a significantly higher number of classes.

Finally, using grid search cross-validation [93], we have performed several sessions of hyper-parameter tuning to identify the most optimal parameters and develop separate XGBoost models for each dataset. The hyper-parameters tuned for XGboost were: booster, eta, n_estimators, max_depth, min_child_weight, gamma, learning_rate, subsample, colsample_bytree, reg_alpha, reg_lamda. Similarly, the k parameter of *k*-NN, was tuned using grid search cross-validation.

**Figure 3:**
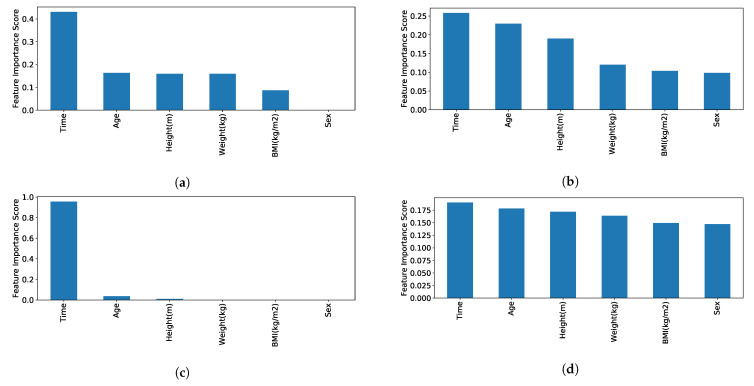
Impurity-based feature importance of the xgboost forests based on mean decrease in impurity as calculated by XGBClassifier.feature_importances_ for (**a**) TUG experiment, (**b**) TUG synthetic, (**c**) FTSTS experiment, and (**d**) FTSTS synthetic.

The feature importance obtained by XGboost classifer after hyper-parameter tuning is presented in Figure 3. Features have clearly different contribution to the prediction in the case of the experimental dataset models and the synthetic models. This is predominantly a result of reducing a bias between male and female participants and removing the original experimental data biases in the correlations of height, weight, and BMI with sex. Additionally, as the synthetic datasets cover a wider variety of ages and conditions, the correlation between the condition and the completion time is reduced compared to the clear correlation between stages of difficulty and completion time in the experimental dataset. Thus, the synthetic dataset led to balancing the features better.

## 4. Hybrid ML Approach for Individualised ART-Driven Rehabilitation

**Figure 4:**
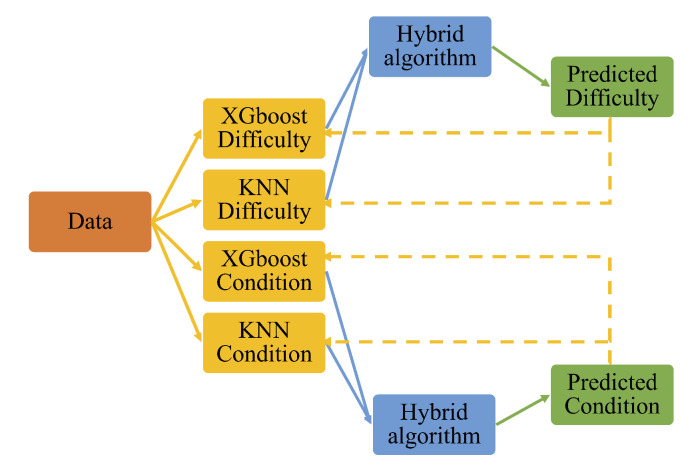
Graphical representation of the hybrid stacking model combining difficulty prediction and condition prediction for a medical test. The data refers to experimental plus synthetic data.

According to the results of the ML method evaluation in the previous section, it is evident that the XGBoost approach demonstrates the highest prediction accuracy among all tested approaches. However, using this model alone does not satisfy the requirements for our proposed system in [1,6]. The scope of the sensory system requires both the monitoring of rehabilitation progress over long periods of time against goals, and the ability to adapt/diagnose a variety of conditions related to stroke survivors such as co-morbidity developed after discharge. Moreover, identifying the areas of difficulty relates to identifying challenges in the user’s daily activities. Thus, combining the models developed for the experiment and/or the conditions is required for each of the two medical tests. Additionally, the combination is necessary to reduce bias and improve variance in the training data. Figure 4 presents the method used to combine the algorithms through the proposed hybrid ensemble learning approach based on the stacking method.

Furthermore, the models must be incrementally re-trainable to allow individualisation. This action also inherently eliminates model bias and importantly improves fairness of the model [94], as it increases user specific biases. Finally, to close the loop with the patient and enable interpretability, and improve reliability in terms of reducing false prediction, a more sophisticated approach is necessary. Here, we propose a method to address the full scope as defined above, proposing an ensemble learning approach based on stacking as presented in Algorithm 2. First, the rate of improvement is calculated. Then, the XGBoost and *k*-NN models are invoked and each returns two predictions—one set for difficulty {$xgb_{prediction}$, $knn_{prediction}$} and one set for condition {$xgb_{prediction}$, $knn_{prediction}$}. The Euclidean distances between each prediction and the healthy cluster are calculated (CalculateEucledianDistance). Finally, based on the calculated rate of improvement and the Euclidean distance, the hybrid algorithm selects the final prediction for difficulty and the final prediction for condition (parameter passed in to ReTrain() is the finalPrediction). These final selections are used to iteratively re-train the XGboost and *k*-NN models.
**Algorithm 2:** Hybrid learning method$time_{completion}\leftarrow ReadSensors\left(\right)$improvement←PolinomialFit(history).get_rate()baseline←MostFrequentPrediction()$xgb_{prediction}\leftarrow XGBoostModel.predict\left(time_{completion}\right)$$knn_{prediction}\leftarrow KNNModel\left(\right).predict\left(time_{completion}\right)$closest,farthest←CalculateEucledianDistance($xgb_{prediction},knn_{prediction},healthy)$**if** improvement=true **then**
 ReTrain(closest) {Closer to Healthy}**else if** improvement=false **then**
 ReTrain(farthest)**else** ReTrain(baseline) {Steady}**end if**userState←stateCalculation(sensorData,improvement,baseline,finalPrediction)

The algorithm uses the rate of improvement for the specific user as an additional feature and combines the XGBoost models (experimental amd synthetic data trained) along with a further semi-supervised model (*k*-NN-based) to establish a final prediction of both the condition and the difficulty faced by the user. The model is incrementally re-trained according to individual performance. The XGBoost prediction returns both the condition class (e.g., stroke, as defined in Section 3.1), and the difficulty class (e.g., difficulty to turn, as defined in Section 3.1). The system is initialised with the condition the user is diagnosed with by a medical professional. If the condition predicted is consistently different to the initialised one, then a co-morbidity may be developing. The algorithm monitors this against the baseline prediction set (baseline) and produces outputs to alert the user to this event.

Additionally, the raw sensor data from the system, the improvement, baseline and final prediction are used to generate a set of user state flags (stateCalculation). These flags are generated to improve the safety of the user of the system and inform carers if necessary. Those are:

- The test starts and does not complete, possible indication of fall.
- Noise and unexpected sensor inputs, possible misuse, sensor faults or multiple users interfering with the test.
- Improvement rate is too high and prediction deviates significantly from baseline, possible indication of user forcing himself (herself) to achieve the goal faster.
- Negative improvement is consistently reported, possible deterioration of user condition.

The *k*-NN models are trained with exactly the same training set as the XGBoost models and incrementally re-trained as in Algorithm 2. Again, a grid search approach was used to tune the *k*-value for each of the four cases. As the *k*-NN is trained in a semi-supervised fashion, it was used in combination with XGBoost to provide a corrective mechanism. Moreover, the *k*-NN method had a better accuracy compared to all other tested models (see Section 5) and was also used with the SMOTE algorithm for dataset balancing.

The goal set in the system is to gradually decrease the completion time that the patient needs to achieve the exercise (goal-oriented rehabilitation). The algorithm is implemented with the parameterisation of the goal in mind so that different levels of improvement rate can be defined to make goals realistic for each individual.

Finally, both the XGBoost and *k*-NN methods have previously been used in IoT applications with real-time design requirements, and the hybrid algorithm does not add statistically significant computational overhead as it amounts to a simple Euclidean distance calculation between the new point and the centroid of the healthy cluster and an if-then-else statement. This is significantly smaller than the third model required for stacking. Thus, the algorithm is appropriate for use in AmI in terms of computational requirements. Additionally, XGBoost has a light computational footprint incremental training method in Python.

### 4.1. ART AI Design Approach

This section discusses the design considerations and ART AI concepts of the proposed method. As identified by [24], we have embedded medical knowledge in the design of the system both through the conditions in the synthetic dataset and the medically approved tests implemented. Additionally to the outputs discussed in the earlier sections, we also generate results demonstrating the inner workings of the algorithm (which model’s output was selected by the hybrid approach, what the *k*-NN and XGboost models predicted individually, which were the other options, and with what probability). This addresses the layered feedback requirement identified in [25] and the exposure of the embedded optimisations of the model as discussed in [24]. All these results can be used as future work by the interface to enable the user to a) understand and b) overwrite the algorithm decisions as suggested in [24,25]. The benefits address both the interpretability and explainability requirements set out in this paper. We have in the previous section discussed the approach for eliminating any bias generated by the system designer leading to fair and non-discriminatory decisions.

We have followed the six design time considerations [43] in the development of the proposed method as follows:

1. Bias of the experimental dataset, in terms of the female/male balance, is mitigated using SMOTE and balanced data between female/male participants through the synthetic dataset. Biases in the remaining features (e.g., age, height, weight) were also addressed as a result of the SMOTE method and the wide variety of sources used in the synthetic dataset;
2. ethical principles followed as presented in [14,42] when designing the datasets and developing the inference algorithms;
3. interdisciplinary team and literature review were significant contributors in the identification of design requirements [1,6], as was also the clear approach to inclusion/exclusion of each paper;
4. data provenance both in terms of input and output is transparent to the user and published at the edge node using open protocols. Both *k*-NN and XGBoost are categorised as transparent by [26] and all the generated model information is also included in the model output. However, for security reasons, only authorised users can view and access both input and output information (system login functionality [1]);
5. the feedback is simplified so that lay people can understand it as presented in [1] and users can inquire further into the factors affecting the decision following the model presented in [27]. This follows the ART AI principles of [24,25];
6. decisions are justified both through the probability results, the person’s rate of improvement and the model’s selection process. Results are available to the user.

In the case of health applications, the value system to be used is one that does not in any case worsen the quality of life or the health of the patient. As a result, the manner in which feedback is presented had to be adjusted to ensure that the user is not urged to always perform a faster exercise but rather focuses on long-term goals. Our proposed method is focused on beneficence by its application specifications (rehabilitation support) and through the goal setting theory approach discussed in [1].

Furthermore, the alerts generated by the system support both the identification of system failures and responsible AI principles. The system generated useful information for the state of the user that can identify user safety and critical events.

## 5. Evaluation and Results

The accuracy, comparison between the proposed hybrid approach, and benchmarks, averaged over all classes, are presented in Table 2. These results justify the combined use of the two models based on accuracy.

To further investigate the reduced performance of the hybrid approach in the TUG experiment dataset trained model (Table 2), we have examined the prediction accuracy in each of the considered classes. Difficulty to *walk* is most often misclassified by XGBoost (60% misclassification as difficulty to *turn* or difficulty to *stand*). This is corrected by *k*-NN in only 17.89% of the misclassified cases. At the same time, however, difficulty to *turn* (28.57% misclassification), difficulty to *stand* and *fast* (0% misclassification for both) have a far better prediction accuracy. Moreover, *normal* is occasionally misclassified as *fast* (57%), but as they are both healthy conditions, this misclassification is not considered further. Similarly for FTSTS, the misclassification was 0% for both difficulty/*slow* and *fast* classes. Additionally, it is demonstrated in Table 2 that the number of data points in TUG synthetic versus FTSTS synth. do not have a significant effect on the accuracy of the hybrid model. It is rather the misclassification of some classes in TUG experiment that have a more profound effect. The reasons behind this misclassification are further discussed in the following section.

**Figure 5:**
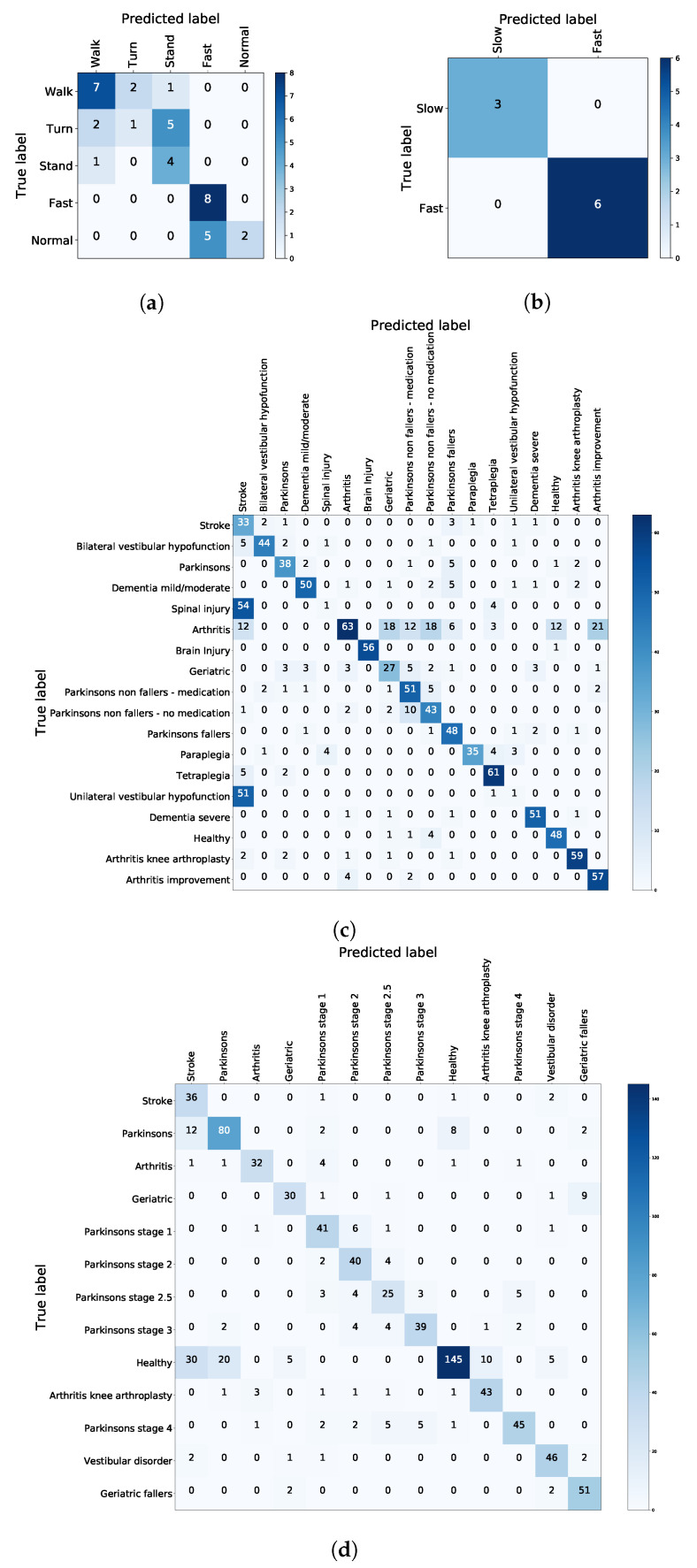
Confusion matrices for the hybrid model presented separately for (**a**) TUG difficulty, (**b**) FTSTS difficulty, (**c**) TUG conditions, and (**d**) FTSTS conditions.

To further investigate the low accuracy of the TUG test, Figure 5a,c demonstrate the confusion matrices of the hybrid model for the difficulties and conditions, respectively. Additionally, the confusion matrices for FTSTS are presented (Figure 5b,d). It is evident that difficulty to turn is often confused with difficulty to stand or difficulty to walk. Moreover, spinal injury and unilateral vestibular hypofunction are often confused with stroke. This explains the relatively low score in TUG accuracy. Furthermore, arthritis is most often misclassified as a range of different conditions. We discus these observations in Section 6. Overall, the specificity, sensitivity, precision, and f${}_{1}$ score for four confusion matrices (corresponding to the four datasets) are presented in Table 3.

In all cases, the hybrid approach has excellent precision and specificity. This is important as false alerts are avoided and do not cause unnecessary alarm to carers. As expected TUG experiment performs lower than other models in sensitivity due to the misclassification between the aforementioned difficulty classes. We consider this to be less problematic as the user is not misclassified as healthy.

**Figure 6:**
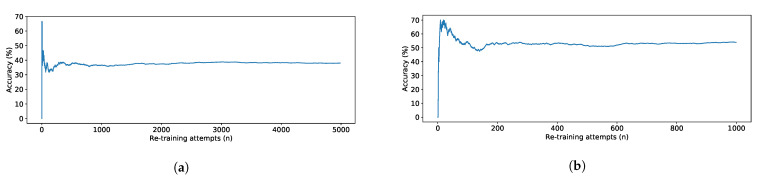
(**a**) Prediction accuracy improvement over time for TUG with difficulty to walk (one of the difficulties that are misclassified in 30% of the cases) and geriatric classification used 5000 times by individual 1, and (**b**) prediction accuracy improvement over time for TUG performed normally (a non-difficulty that is misclassified as *fast* in 71% of the cases) 1000 times by individual 3 classified as *dementia severe*.

The accuracy of the proposed combined approach not only demonstrated improved outcomes (Table 2), but it improves over time as presented in Figure 6. The graphs display the prediction accuracy cumulatively calculated over the full history of use. As demonstrated, the number of correct predictions increases over time resulting in a gradual increase in prediction accuracy. The graphs demonstrate that the system’s behaviour is similar to that of a positive feedback loop in control theory. This is an expected result as the model is incrementally re-trained in a similar manner. This will continuously improve the sensitivity of the model to the specific individual. Moreover, as the user continues to use the system, a possible false negative in a single use will be counterbalanced by true positives/negatives over the lifetime of the system.

For TUG test, the accuracy of the hybrid approach is 57.89%, which is an improvement from the original 42.5% for the same dataset using the XGBoost model alone. Similarly for FTSTS, the accuracy of the hybrid approach was 100%, which is an improvement over the original value of 70%.

Furthermore, we have investigated the most frequently predicted condition for each of the TUG and FTSTS experimental dataset classes. The results are presented in Table 4. These results demonstrate the validity of the claims in [1], where the simulated experimental conditions were related to patient and elderly subject completion times.

We have performed similar tests for each participant of the experiment and for each simulated difficulty. The prediction accuracy improved or stayed the same over time in 81.82% of the tested cases for the test set. Accuracy improved over time in 100.0% of all the tested combinations of {individual, difficulty} in the full set combining test and train data.

## 6. Discussion

In this section, we discuss the suitability of the proposed method for individualised patient-centric home-based rehabilitation. Based on the results, the proposed method is capable of providing accurate home-based rehabilitation support, with improvement of individualisation in terms of accuracy over time based on observations of a specific individual. The method is suitable for both rehabilitation goal setting as well as diagnosis of co-morbidities.

An important observation is the feature importance of the XGBoost model, which is evidently different for each of four datasets. In the case of difficulties in the experimental dataset for both TUG and FTSTS tests, time of completion clearly has a higher importance compared to other features. However, in terms of conditions (synthetic dataset), all features are contributing in some cases almost equally (refer to Figure 3). The high importance of the time of completion feature in the experimental dataset is expected beause of the bias presented in the dataset due low variability in terms of other features (e.g., all participants belong to the same age group and only one was female).

In the confusion matrices, we have identified the most often misclassifications of the hybrid model. We attribute these to the use of a small amount of sensors described in [1]. With the use of a small amount of sensors, the different stages of the TUG are not supplied as separate features. Instead, the full time of completion of the TUG is the used feature. This results in the model’s inability to clearly differentiate between the two difficulties of *turn* and *stand*. We argue that an additional sensor for FTSTS would mitigate this issue. Further work will be needed to explore the use of additional sensors and their effect on reducing misclassification between the two difficulties but also the effect on the acceptance criteria. Another possible solution to avoid the use of additional sensors would be to break down the TUG completion time to separate stages. This would avoid increasing the complexity of the system or the cost. Moreover, the stages would become additional features for the difficulty prediction model. Thus, this option may offer better alignment with the acceptance criteria. However, further work will be needed to investigate the effect on misclassification and accuracy.

Furthermore, we have identified that two conditions in TUG are always misclassified as *stroke*. We believe this is due to the similarity of the effect of the condition on the walking pattern. For example, a severe stroke could result in severe vestibular hypofunction or have similarities to spinal injury due to the effect on the motor control system. Moreover, we have not differentiated between mild, moderate, or severe stroke symptoms. Further work can explore the refinement of the dataset or further feature selection approaches for improving classification of the severity of stroke in mild, moderate, and severe. This could improve performance of the hybrid model. A similar effect is seen in the case of *arthritis*, where various stages of arthritis are not differentiated. On the contrary, the differentiation between stages of dementia and Parkinson’s have proven to have better performance as evident from the confusion matrices in both TUG and FTSTS.

In terms of FTSTS, the *difficulty/slow* class is never confused with the *fast* class. However, the *healthy* class is rarely misclassified as *stroke* (Figure 5b,d). As before, we hypothesise that a fine grained presentation of stroke stages in the dataset would address this issue. Further research would be required to investigate this hypothesis. It is possible that mild cases or early stroke signs are misclassified as *healthy*. This issue should be addressed in future work.

Though a direct comparison with other ML-based approaches used for FTSTS and TUG tests cannot be made due to unavailability of common datasets and minor differences in classification of test stages, by analysing the accuracy results reported in the literature, we conclude that the proposed hybrid model achieves acceptable accuracy that surpasses the results reported in the literature. Indeed, the hybrid model reports higher accuracy than state-of-the-art AI methods that use intrusive means of monitoring such as cameras or the Kinect sensor. For example in [40] FTSTS decision tree models and *k*-NN have demonstrated 92% and 91% accuracy, respectively. In [41], the accuracy of the proposed classifier was 94.68% for FTSTS, and a review of similar publications demonstrates that this is the state of the art for FTSTS AI models. Similarly, in [39], accuracy of all assessed classifiers including NNs did not exceed 85% for TUG. As a result, our proposed hybrid approach improves accuracy over and above the state of the art for both tests while also addressing a series of constrains such as computational requirements, incremental re-training, and ART AI. Moreover, we further address all the criteria in [6] to enhance engagement and motivation.

As identified by our above analysis, an open problem that remains for the research community to address is the creation of common publicly available datasets to enable further studies in this space and provide a basis for comparison of different techniques. As discussed in Section 3, synthetic datasets are slowly becoming more common in the medical domain to address this problem and work around the issue of revealing private information. However, no benchmark datasets, synthetic or otherwise, are yet established.

## 7. Conclusions

In this paper, we have presented a hybrid learning approach for patient-centric individualised home-based rehabilitation support considering unbiased, explainable, and interpretable AI. To address unbiased AI requirements, we presented a method for generating synthetic datasets to complement experimental observations. We then evaluated supervised learning algorithms for suitability where XGboost and *k*-NN were identified as the most suitable candidates in terms of accuracy, computation footprint, and explainability. Finally, we presented a hybrid ML algorithm combining ensemble learning and hybrid stacking using XGBoost and *k*-NN to maintain a low computation footprint. We discussed the ART AI design time considerations and output in respect to explainability and interpretability. Finally, we presented the individualisation approach which follows constant re-training of the hybrid model.

We demonstrated that the synthetic dataset meets the ART AI requirements, as it is based on clinical observation and improves the contribution of all features in the model. The final model reaches up to 100% accuracy for FTSTS in both the prediction of difficulty and the prediction of associated patient condition. In the case of TUG, the performance reaches up to 100% in the prediction of patient condition and 83.13% in the prediction of area of difficulty. This surpasses the results reported in the literature for TUG and FTSTS test with state-of-the-art AI classifiers by 5% and 15%, respectively. Misclassification occurs between the difficulty to turn and difficulty to stand cases. Normal TUG tests are confused with fast in 57% of the cases. The re-training element incrementally improves overall model accuracy, which means that as the user continues rehabilitation at home, the device better adapts to the user’s specific difficulty areas and conditions. The confusion matrices imply that some of the patient conditions such as stroke and arthritis are largely generalised classes, and if broken down by severity, the model could improve. Furthermore, to better differentiate between difficulty to turn and difficulty to stand, the TUG completion time feature could be split into two additional features for individual stages of the test.

### Future Work

In this paper, we have presented the hybrid ML model approach as well as the input and output data generated to support ART AI. In the future, we will further develop the user interface. We aim to follow the dialogic XAI model presented in [27] in combination with state-of-the-art human–computer interaction (HCI) and cognitive theory approaches for ART AI as discussed in [95]. Moreover, we aim to address the misclassification issues discussed in this paper by improving the feature construction and feature selection of more fine-grained stroke severity and difficulty level of turn and stand classes, possibly by augmenting sensor data or creating common publicly available datasets to enable further studies in the space, enable reproducibility, and compare different techniques.

## Figures and Tables

**Table 1:** Fivefold cross-validation accuracy results of ML models. Accuracy is calculated as the fraction of correct predictions using sklearn.metrics.accuracy_score.

Model	TUG Experiment	TUG Synthetic	FTSTS Experiment	FTSTS Synthetic
SVM	0.3099	0.3921	0.9778	0.3625
Regression	0.35	0.0605	1	0.0705
*k*-NN (*k* = 5)	0.475	0.7006	0.6917	0.6791
XGboost before hyperparam tuning	0.4600	0.7667	0.9778	0.7827
XGboost after hyperparam tuning	0.65	0.8006	1	0.7965

**Table 2:** Accuracy results of XGBoost, *k*-NN, and hybrid models using the test dataset.

Model	TUG Experiment	TUG Synthetic	FTSTS Experiment	FTSTS Synthetic
xgb 5-f	0.625	0.6052	0.7	0.577
knn 5-f	0.45	0.5932	0.7	0.5930
hybrid 5-f	0.5789	1	1	1

**Table 3:** Hybrid model confusion matrix metrics.

Metric	TUG Experiment	TUG Synthetic	FTSTS Experiment	FTSTS Synthetic
Sensitivity	0.33	0.9	1	0.87
Specificity	0.78	0.94	1	1
Precision	0.78	0.94	1	1
f${}_{1}$ score	0.47	0.92	1	0.93

**Table 4:** Hybrid model most frequent association of difficulty and condition for the experimental test dataset of the eight participants.

Test	Cluster	Condition
TUG	Difficulty to Walk	Geriatric
	Difficulty to Turn	Paraplegia
	Difficulty to Stand	Dementia severe
	Normal	Arthritis
	Fast	Unilateral vestibular hypofunction
FTSTS	Difficulty	Vestibular disorder
	Fast	Parkinson’s stage 4
